# Experimental warming causes large yield reduction of spring highland barley, and the changes of the phyllosphere microbial community represents the extrinsic manifestation of the underlying mechanism

**DOI:** 10.1371/journal.pone.0319612

**Published:** 2025-04-29

**Authors:** Zhiming Zhong, Guangyu Zhang, Gang Fu

**Affiliations:** Lhasa Plateau Ecosystem Research Station, Key Laboratory of Ecosystem Network Observation and Modeling, Institute of Geographic Sciences and Natural Resources Research, Chinese Academy of Sciences, Beijing, China; National Institute of Agricultural Research - INRA, MOROCCO

## Abstract

Changes in phyllosphere microbial communities of highland barley caused by climate warming (e.g., increases in the abundance of pathogenic fungi) is a potential important mechanism leading to the decrease in yield of highland barley. However, there are no reports that the loss of highland barley yield caused by climate warming is attributed to changes in phyllosphere microbial community of the highland barley. Here, based on a field warming experiment in the Lhasa city, Tibet, we examined the responses of phyllosphere bacterial and fungal communities to warming and their feedbacks to the yield of spring highland barley. Warming decreased yield of highland barley by 34.04%, but increased soil temperature by 2.40°C, phyllosphere fungi species richness by 50.00%, fungi Chao1 by 37.55%, fungi phylogenetic diversity by 51.74%, and pathotroph fungi by 529.17%. Yield of highland barley decreased with increasing phyllosphere fungi species richness, fungi Chao1, fungi phylogenetic diversity and pathotroph fungi. Warming altered phyllosphere bacterial functional composition, which was also marginally correlated with yield of highland barley. Moreover, warming only caused the forward shift of each phenology, and did not change the time interval between the two adjacent phenological periods. The change of phyllosphere microbial community, especially fungal community, caused by warming is a potentially important mechanism leading to the yield reduction of highland barley, which provides a new perspective for the regulatory mechanism of highland barley yield reduction and even grain yield reduction under future climate warming. More importantly, the scientific findings of this study may provide potential new directions (e.g., exogenous addition of beneficial phyllosphere microbial fertilizers) on how to mitigate grain yield reduction caused by climate warming.

## 1 Introduction

The human population on Earth is growing rapidly and is expected to exceed 9 billion by 2050 [1]. Feeding a growing population is a major challenge for global food production. To address this, global food production needs to increase by 70% to meet demand by 2050 [2]. However, climate warming poses a serious threat to global food production [3,4]. The increasing global population and climate warming are both serious threats to global food security. Therefore, figuring out what measures should be taken to address the issue of food production reduction resulting from climate warming has already become a major and inescapable challenge for all mankind. An in-depth exploration of the internal regulatory mechanism underlying food production reduction due to climate warming is a prerequisite for alleviating the predicament of such reduction. At present, it is generally believed that the grain reduction caused by climate warming is related to the shortened growth period of grain crops and the inhibition of physiological and biochemical indexes such as photosynthesis [5]. Many studies have shown that there are co-evolution relationships between food crops and their phyllosphere microorganisms [6–10]. Meanwhile, experimental warming has been proven to cause changes in the phyllosphere microorganisms of plants, including a possible increase in phyllosphere pathogenic fungi and a decrease in phyllosphere symbiotic fungi [11,12]. In turn, these changes in phyllosphere microorganisms are very likely to have a series of adverse effects on host plants [11]. All these suggest that changes in phyllosphere microbial communities of food crops under the background of climate warming (e.g., climate warming may increase the abundance of potential pathogens in the phyllosphere) may be an important underlying mechanism leading to grain yield reduction. However, given the extreme paucity of existing research on how phyllosphere microbial communities of food crops respond to climate warming (with fewer than 10 relevant papers published worldwide) [11,13–15], there remains substantial uncertainty regarding whether and how climate warming will alter the structure of such communities. Therefore, in order to solve the scientific problem of whether the grain yield reduction caused by climate warming is related to the changes in the phyllosphere microbial communities caused by climate warming, it is necessary to further explore the effects of climate warming on phyllosphere microbial communities of food crops. Therefore, strengthening the research on the change rules of phyllosphere microbial communities of food crops and its impact on food yield is conducive to the development of new theories, new methods and new tools related to global food production under the background of climate warming.

Highland barley is the main crop in Tibet and the main food of Tibetan compatriots, and its yield is seriously threatened by climate warming (1°C warming can result in 10.55% decline in the yield of highland barley, and the maximum yield decreases by more than 30%) [4,16,17], which is unfavorable to the highland barley security and food security of Tibet and even the stability of the border area. There are only a few studies (<30) on the effects of experimental warming on the alpine farmland ecosystem of the Qinghai-Tibet Plateau [5,18]. These studies have not examined responses of alpine crop phyllosphere microbial communities to climate warming and their impacts on food crop yields. This not only limits our in-depth understanding of changes of phyllosphere microbial communities in the highland barley under the background of climate warming, but also limits our in-depth exploration of potential beneficial phyllosphere microbial resources in the highland barley. Therefore, it is necessary to strengthen the research on changes of the phyllosphere microbial communities of highland barley under the background of climate warming and their impacts on the yield reduction of highland barley, so as to provide data support and theoretical basis for alleviating the yield reduction of highland barley caused by climate warming. It will provide scientific and technological support for the comprehensive and high-quality development of Tibet Autonomous Region in social, economic and ecological aspects under the premise of ensuring the safety and food security of Tibetan highland barley.

In this study, relying on a field warming experiment, we probed into the responses of phyllosphere bacterial and fungal communities and the yield of highland barley to warming and their interrelationships in the Tibet. This study might offer a novel underlying mechanism regarding the reduction of barley yield and even cereal crop yield in the context of climate warming. We hypothesized that warming can lead to a reduction in highland barley yield, which may have an apparent correlation with the increase in phyllosphere pathogenic microbes and the decrease in symbiotic microbes of highland barley caused by warming.

## 2 Materials and methods

### 2.1 Experiment design and variables measurements

The experiment platform was set up in 2015 and the time node of this study was 2018. This experiment set up four warming treatment methods, specifically as follows. The first one was the control (CK), implying no warming throughout the year. The other three treatments involved warming during the highland barley growing season but not during the non-growing season. They included daytime warming (from 8:00–20:00 Beijing time), nighttime warming (from 20:00–8:00 Beijing time), and all-day warming (from 8:00–8:00 the next day Beijing time). Each experimental warming treatment was set with four replicates to ensure the reliability and accuracy of the experimental data. The area of each experimental plot was uniformly set at 2 meters by 2 meters, and the distance between any two experiment plots was maintained at more than 5 meters, effectively reducing the possible mutual influence between plots and further ensuring the independence and accuracy of the experimental data. During the spring highland barley growth period from 2015 to 2018 (from April 23 to August 24 in 2015; from April 15 to August 16 in 2016; from April 15 to August 18 in 2017; from April 15 to August 23 in 2018), in order to effectively increase the soil temperature during the growing season of highland barley, a special infrared heater (produced by Kalglo Electronics Inc., Bethlehem, Pennsylvania, USA) was used. The heater was suspended at a height of approximately 1.70 meters. During the entire growth period of highland barley, for the three warming treatment methods of daytime warming, nighttime warming, and all-day warming, the output power of each infrared heater was stably maintained at 2000 watts, thereby providing stable and comparable warming conditions for the experiment. With the help of the HOBO microclimate station (produced by Onset Computer, Bourne, Massachusetts, USA), the soil temperature (*T*_s_) and soil moisture (SM) at a depth of 5 centimeters were accurately measured. Among them, during the entire spring highland barley growing season in 2018, the daytime warming, nighttime warming, and all-day warming increased the soil temperature by 0.37°C, 1.73°C, and 2.40°C (**S1** Fig), respectively. In the sowing process, the row spacing of highland barley sowing was approximately 0.25 meters, and the sowing amount of highland barley was precisely controlled at 18.75 grams per square meter. At the same time, in terms of fertilization, operations were carried out in two key periods. Before sowing each year, a rotary cultivator was used to till the experimental area to a depth of 20 centimeters, creating a good soil physical environment for the growth of highland barley. Then, approximately 18.00 grams per square meter of diammonium phosphate and 12.00 grams per square meter of urea were applied to each experimental warming plot. Between the jointing stage and the heading stage, 9.00 grams per square meter of diammonium phosphate and 6.00 grams per square meter of urea were applied again to meet the nutrient requirements of highland barley in different growth stages and ensure the consistency and controllability of the plant growth environment during the experimental process.

However, only the CK and all-day warming treatments were used in this study. We recorded the phenological period of highland barley (i.e., seeding period, trefoil period, tillering period,jointing period, booting period, heading period, flowering period, pustulation period and mature period). More than 50% of highland barley plants reaching the corresponding phenological period was treated as the criterion [16]. During the jointing period of highland barley, we collected leaves of highland barley in the control and warming (24 h each day) treatments, which were used to measure phyllosphere bacterial and fungal communities. The barcoded primers of 5′-ACTCCTACGGGAGGCAGCA-3 (i.e., 338F) and 5′-GGACTACHVGGGTWTCTAAT-3′ (i.e., 806R) were used to amplify 16SrDNA V3-V4 region for bacteria. The barcoded primers of 5′-GCATCGATGAAGAACGCAGC-3′ (i.e., ITS3-F) and 5′-TCCTCCGCTTATTGATATGC-3′ (i.e., ITS4-R) were used to amplify ITS2 region for fungi [12]. Bases in the sliding window were used to calculate the average quality value, and then the non-conforming sliding window was cut directly. Operational taxonomic units (OTU, default 97% similarity) were introduced in the process of amplicon sequencing analysis. The UPARSE method was used for OTU clustering. Bacterial species identification was based on the silva (v132) database. Fungi species identification was based on the unite (v8.0) database. We collected soils at the depth of 0–5, 5–15, 15–25 and 25–35 cm during the end of growing season of highland barley in 2018. Soil organic carbon (SOC), total nitrogen (TN), total phosphorus (TP), total potassium (TK), available nitrogen (AN), available phosphorus (AP), available potassium (AK) and pH were measured. At the end of growing season of highland barley, we measured the yield of highland barley. Soil temperature and moisture at 5 cm depth were monitored by HOBO stations [19].

### 2.2 Statistical analyses

We obtained taxonomy α-diversity variables (i.e., species richness, Chao1, ACE, Shannon and Simpson), functional α-diversity variables (i.e., function number, Chao1, ACE, Shannon and Simpson) and phylogenetic α-diversity variables (i.e., phylogenetic diversity, PD; mean pairwise distance, MPD; mean nearest taxon distance, MNTD) [20,21]. We used principal coordinate analysis to examine whether there were differences of community composition between the control and warming treatments, based on some previous studies [22]. *T*-test was used to examine warming effects on the yield of highland barley, α-diversity, fungal abundance of functional groups (i.e., pathotroph fungi, saprotroph fungi and symbiotroph fungi), and soil variables. Univariate regression analyses were used to obtain the relationships between yield of highland barley and independent variables. All statistical significances were at least at the level of *p*<0.05.

## 3 Results and discussion

### 3.1 Highland barley yield and phenology

**Fig 1:**
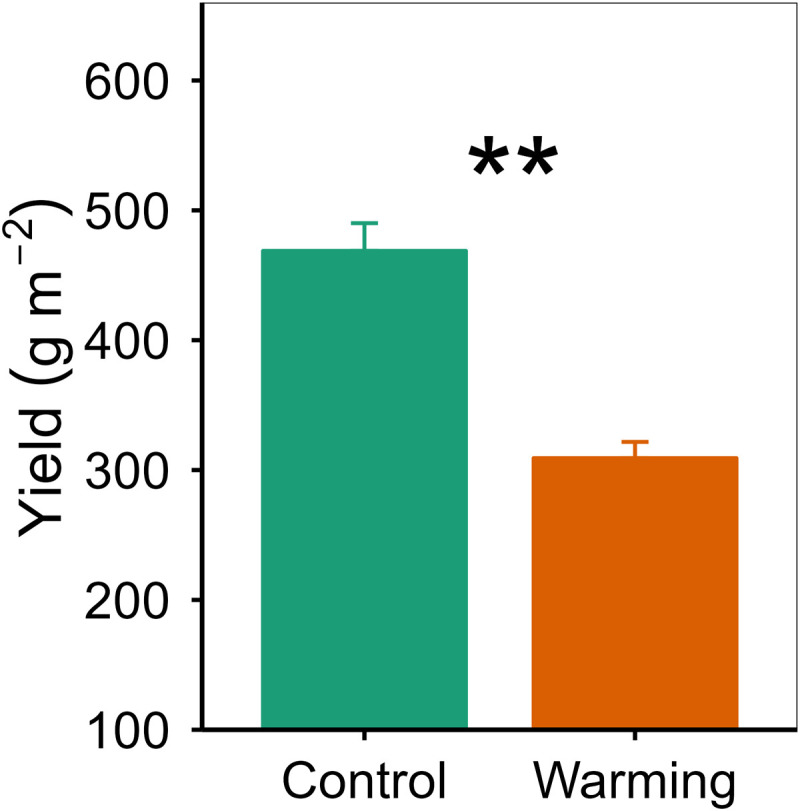
Comparison of yield between the control and warming treatments (mean±se). ** indicates *p*<0.01.

Warming decreased yield of spring highland barley by 34.04% (Fig 1), which was comparable to warming-induced decreased magnitude (31.35%–61.19%) in the yield of spring highland barley reported by previous studies [16,17]. However, the phenological period of spring highland barley moved forward by 2 days under the warming conditions, and there were no changes in the number of days between the two adjacent phenological periods (**S1 Table**). These findings implied that warming-induced phenological changes did not fully explain the decrease in the yield of spring highland barley caused by warming. Therefore, there should be more important underlying mechanisms for the yield reduction of highland barley caused by warming.

### 3.2 Soil nutrients

In line with some previous studies [23,24], warming did not alter soil organic carbon and total nitrogen (**S2** Fig). Warming also did not alter soil available nutrients, pH and moisture (**S1**–**S2** Fig). These implied that warming did not cause adverse soil physical and chemical properties to the growth and yield of spring highland barley (e.g., warming did not cause soil acidification or soil alkalization). That is, the negligible changes of soil physical and chemical properties caused by warming were unlikely to be the main reason for the yield reduction of spring highland barley under warming conditions. Moreover, some previous studies demonstrated that warming did not alter soil fungal and bacterial communities of spring highland barley systems [19,25,26]. These findings implied that the negligible effects of warming on soil fungal and bacterial communities were also unlikely to be the main driver of spring barley yield reduction under the warming conditions.

### 3.3 Phyllosphere microbes

**Fig 2:**
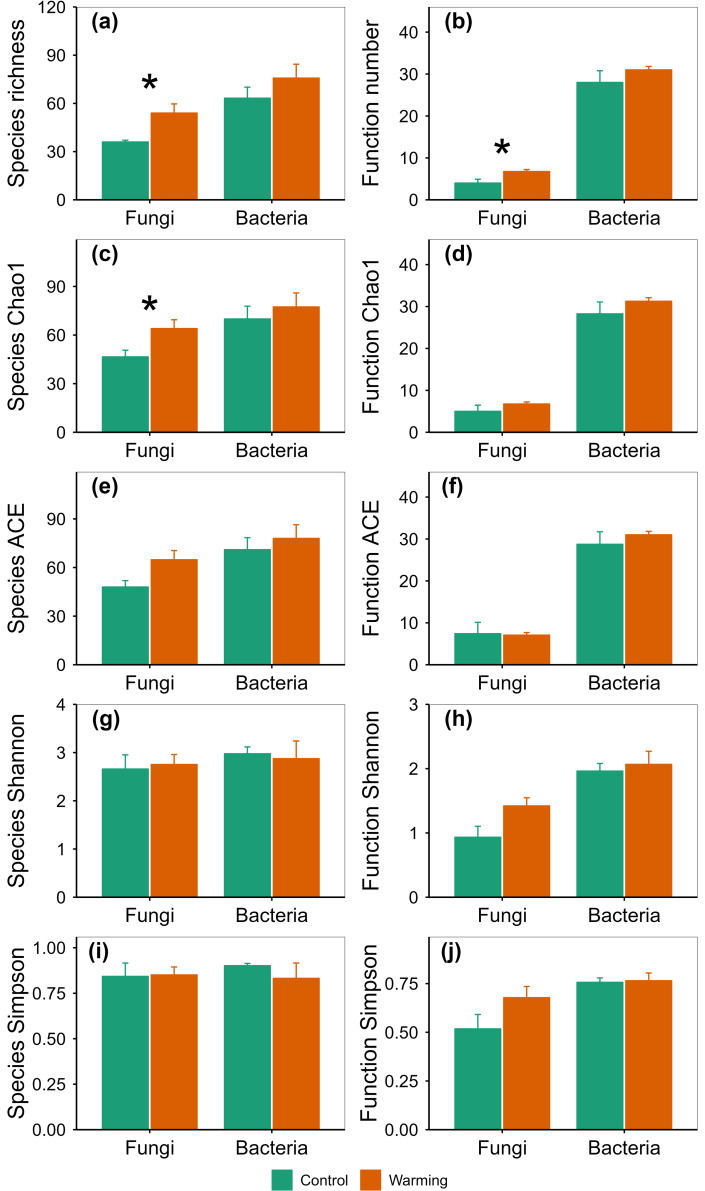
Comparison of species and functionα-diversity of phyllosphere fungi and bacteria between the control and warming treatments (mean±se). * indicates *p*<0.05.

**Fig 3:**
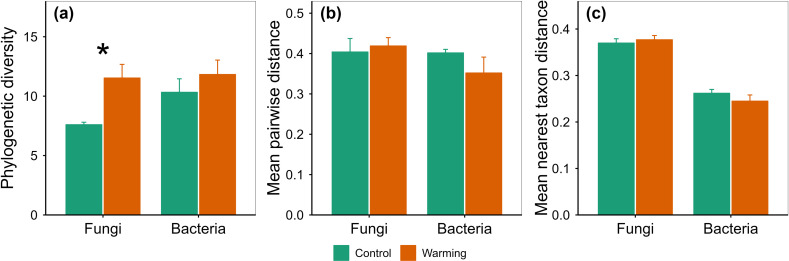
Comparison of phylogeneticα-diversity of phyllosphere fungi and bacteria between the control and warming treatments (mean±se). * indicates *p*<0.05.

**Fig 4:**
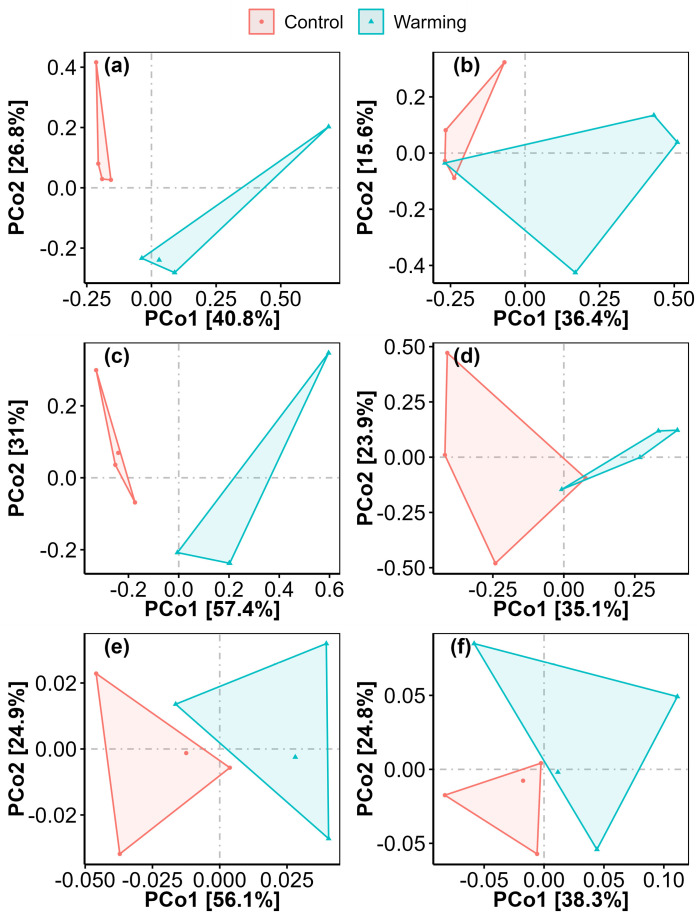
Comparison of community composition of phyllosphere bacteria (a, c, e) and fungi (b, d, f) at species (a, b), function (b, d) and phylogenetic (e, f) levels between the control and warming treatments.

**Fig 5:**
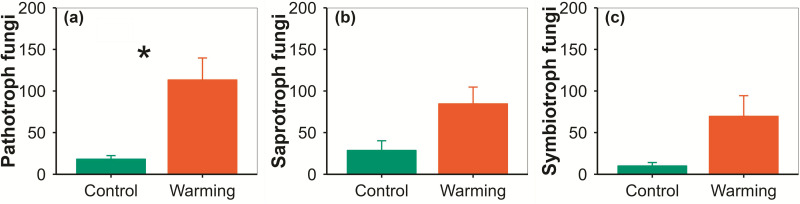
Comparison of phyllosphere pathotroph fungi (a), saprotroph fungi (b) and symbiotroph fungi (c) between the control and warming treatments (mean±se). * indicates *p*<0.05.

**Fig 6:**
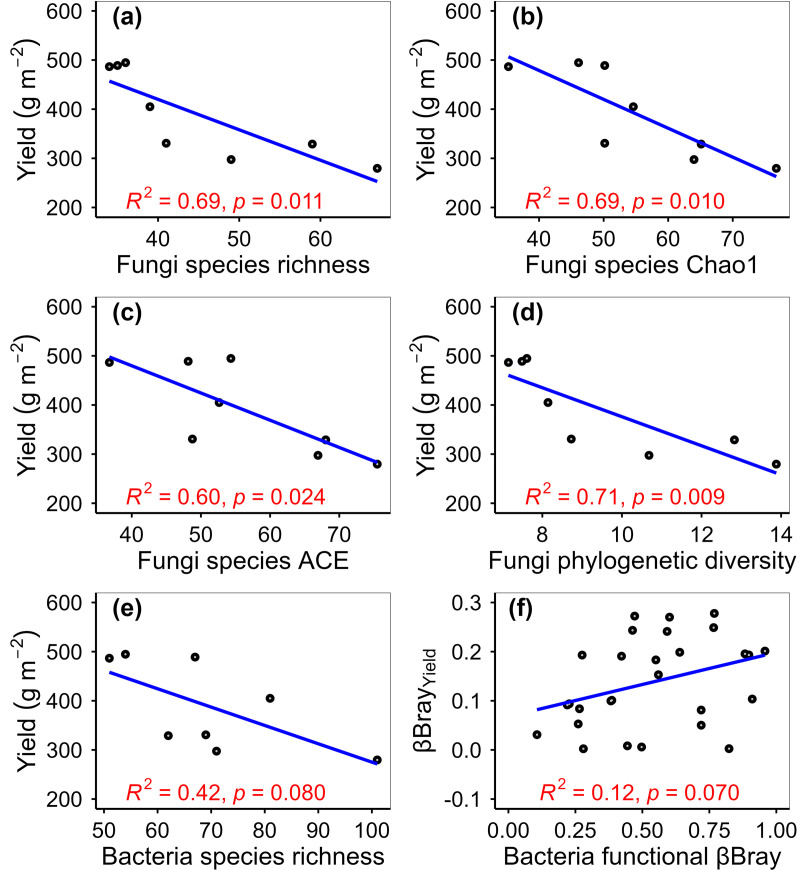
Relationship of yield with phyllosphere fungi species richness (a), fungi species Chao1 (b), fungi species ACE (c), fungi phylogenetic diversity (d), and bacterial species richness (e), and relationship between theβBray values of yield and βBray values of functional composition.

**Fig 7:**
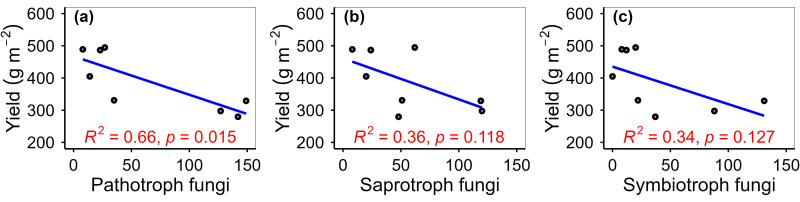
Relationship of yield with phyllosphere pathotroph fungi (a), saprotroph fungi (b), and symbiotroph fungi (c).

Warming increased phyllosphere fungi species richness by 50.00%, fungi Chao1 by 37.55%, fungi function number by 68.75%, and fungi phylogenetic diversity by 51.74% (Figs 2–3). Warming altered phyllosphere bacterial community composition at species, function and phylogenetic levels, and fungal community composition at phylogenetic level (Fig.4). Warming increased phyllosphere pathotroph fungi by 529.17% (Fig.5), which was similar with some previous studies conducted in alpine grasslands of the Tibetan Plateau [12]. Meanwhile, yield of spring highland barley decreased with increasing phyllosphere fungi species richness, Chao1 and ACE, fungi phylogenetic diversity and pathotroph fungi (Figs.6–7). All these findings suggested that the changes in the functional composition of phyllosphere bacteria induced by warming, especially the increases in the α-diversity of phyllosphere fungi and pathotroph fungi, may be causal mechanisms for the yield reduction of spring highland barley. Therefore, the application of beneficial phyllosphere microbial fertilizers or inhibitors against harmful phyllosphere microorganisms was suggested during the highland barley cultivation under future climate warming. These approaches may provide a means to relieve the yield reduction predicament of highland barley induced by climate change and pioneer a novel avenue for safeguarding the highland barley yield. This suggestion was supported by previous studies. For example, previous studies have shown that phyllosphere probiotics can promote seed germination, plant growth, disease resistance and yield of grain crops [27–29]. Moreover, the effects of grain yield increase achieved by regulating the phyllosphere microbial community of grain crops can be equal to that achieved by applying chemical fertilizer [30].

As a key driving factor, climate warming triggers a chain reaction of ecological environment parameters, deeply disturbing the stable state of the symbiotic system between highland barley and the phyllosphere microbial community. It dominates the succession trajectory of the phyllosphere microbial community and breaks the original symbiotic equilibrium [11]. The warming phenomenon leads to an increase in the temperature of highland barley leaves, accelerates the metabolic turnover rate of the leaves, enhances the exudation and dissolution flux of nutrients on the leaf surface, and remodels the dynamic of temperature and humidity and gas exchange parameters in the phyllosphere [31]. The adaptive adjustment of the phyllosphere microbial community exhibits multi-dimensional characteristics, covering hierarchical changes such as individual metabolic disorders, alterations in the pattern of population competition and symbiosis, and the alienation of community functions [32]. The growth of cold-adapted microbial populations is inhibited, while the proliferation trend of mesophilic and thermophilic microbial groups is enhanced. Under extreme high-temperature conditions, the ecological niche structure of the phyllosphere microbial community is imbalanced, and opportunistic microorganisms multiply, driving the in-depth reconstruction of the community composition and functional characteristics [33]. After the imbalance of the phyllosphere microbial community, it impacts the key growth links of highland barley in multiple dimensions through physiological damage, nutrient limitation, and signal perturbation, such as a decrease in photosynthetic efficiency, a disorder in respiratory rhythm, and an inaccurate stress response. The population of pathogenic fungi expands out of control [34]. Taking the sharp increase in the population of pathotrophic fungi in this study as an example (**Fig.5**), it destroys the synthesis process of photosynthetic pigments in leaves, blocks the nutrient transport channels in vascular bundles, and triggers excessive energy consumption in plant defense responses by means of hyphal invasion and toxin release, resulting in a lack of accumulation of photosynthetic products [35]. The scale of the symbiotic beneficial microbial community is reduced, weakening the functions of nutrient activation, fixation, and targeted transport. The activity of nitrogen-fixing bacteria in the phyllosphere is inhibited, and the input of nitrogen fixation is drastically reduced. The growth of plants is deeply trapped in a nutrient-deficient situation, and the lack of source materials restricts the potential for yield growth [36]. The variation of the microbial metabolite profile leads to the secretion of growth-inhibiting substances or the disturbance of hormone signal transduction pathways, causing a disorder in the flowering time sequence, a sharp decline in the seed setting rate, and poor seed plumpness, severely damaging the yield formation process from the key nodes of reproduction. Therefore, after the initiation of climate warming, through the dynamic transmission of the phyllosphere microbial community, obstacles are successively constructed at the levels from the supply of photosynthetic raw materials, the nutrient distribution network to the reproductive growth program. All links work in coordination and jointly shape a coherent causal logic chain, leading to a decline in highland barley yield. It reversely confirms the indirect negative effect of climate warming, closes the causal logic loop of “climate warming-phyllosphere microbial variation-yield decline”, and aggravates the risk of food security in the agricultural ecosystem.

### 3.4 Uncertainties

There were still some uncertainties in this study. Both the yield of highland barley and phyllosphere microorganisms may show interannual fluctuations, and the phyllosphere microorganisms may also have seasonal variation characteristics. However, in this study, the phyllosphere microorganism observation was only carried out for one year and during only one phenological period in one site, and the changes in the physiological and biochemical characteristics of highland barley leaves were not directly observed. Therefore, it was necessary to strengthen relevant scientific research in the future to verify the preliminary conclusions in this study.

## 4 Conclusions

In conclusion, the variations in the phyllosphere microbial community as a result of warming ostensibly clarify the phenomenon of the decrease in the yield of spring highland barley caused by warming. This finding may be a new underlying mechanism for yield reduction of spring highland barely under climate warming. The findings of this study suggest that it may be an effective method to weaken the effects of climate warming-induced yield reduction of highland barley by artificially adding beneficial phyllosphere microbial fertilizers or inhibitors of harmful phyllosphere microorganisms, etc.

## Supporting information

S1 Table Mean days in advance of phenological period caused by warming (days) .
(DOCX)

S1 Fig Comparison of soil temperature (a), and soil moisture (b) between the control and warming treatments. (DOCX)

S2 Fig Comparison of soil organic carbon (SOC) (a), total nitrogen (TN) (b), total phosphorus (TP) (c), total potassium (TK) (d), available nitrogen (AN) (e), available phosphorus (AP) (f), available potassium (AK) (g) and pH (h) between the control and warming treatments. (DOCX)
