# Supplementary material for: Experimental warming causes large yield reduction of spring highland barley, and the changes of the phyllosphere microbial community represents the extrinsic manifestation of the underlying mechanism
Source: PLoS One. 2025 Apr 29;20(4):e0319612. doi: 10.1371/journal.pone.0319612 (PMC12040170; doi:10.1371/journal.pone.0319612)
Supplement: S1 Table — . (DOCX) [file pone.0319612.s001.docx]

**Table S1** Mean days in advance of phenological period caused by warming (days).

| Seeding period | Trefoil period | Tillering period | Jointing period | Booting period | | Heading period | Flowering period | Pustulation period | | Mature period |
| --- | --- | --- | --- | --- | --- | --- | --- | --- | --- | --- |
| –2 | –2 | –2 | –2 | –2 |  | –2 | –2 | –2 |  | –2 |
