# Supplementary material for: Experimental warming causes large yield reduction of spring highland barley, and the changes of the phyllosphere microbial community represents the extrinsic manifestation of the underlying mechanism
Source: PLoS One. 2025 Apr 29;20(4):e0319612. doi: 10.1371/journal.pone.0319612 (PMC12040170; doi:10.1371/journal.pone.0319612)
Supplement: S1 Fig — (DOCX) [file pone.0319612.s002.docx]

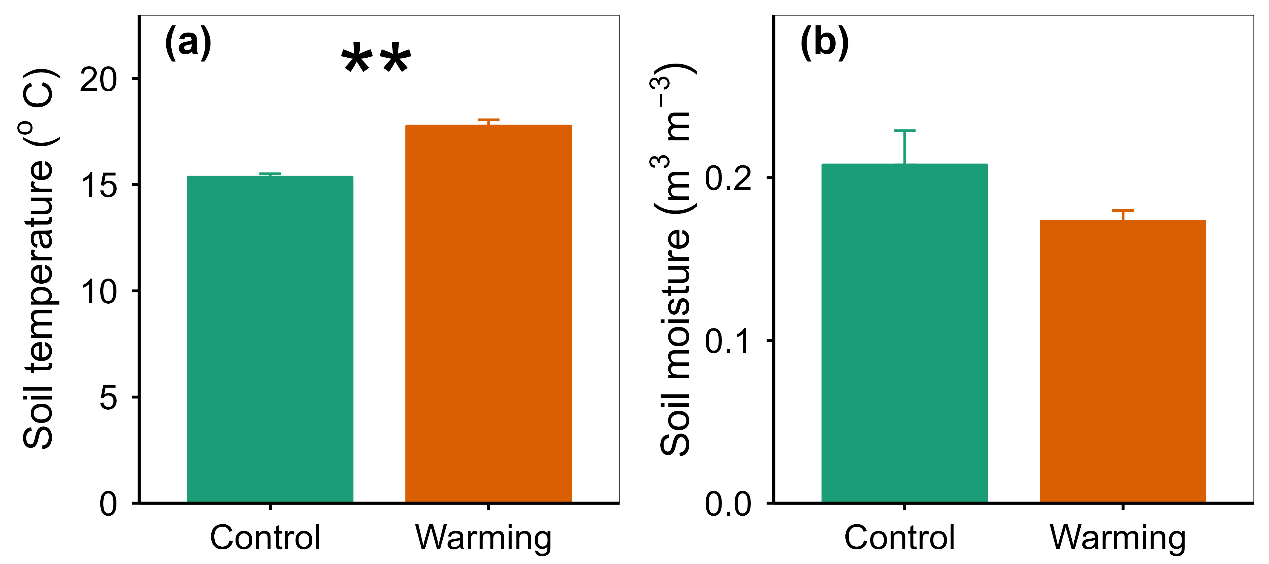


**Fig.S1**. Comparison of soil temperature (a), and soil moisture (b) between the control and warming treatments.
