# Supplementary material for: Experimental warming causes large yield reduction of spring highland barley, and the changes of the phyllosphere microbial community represents the extrinsic manifestation of the underlying mechanism
Source: PLoS One. 2025 Apr 29;20(4):e0319612. doi: 10.1371/journal.pone.0319612 (PMC12040170; doi:10.1371/journal.pone.0319612)
Supplement: S2 Fig — (DOCX) [file pone.0319612.s003.docx]

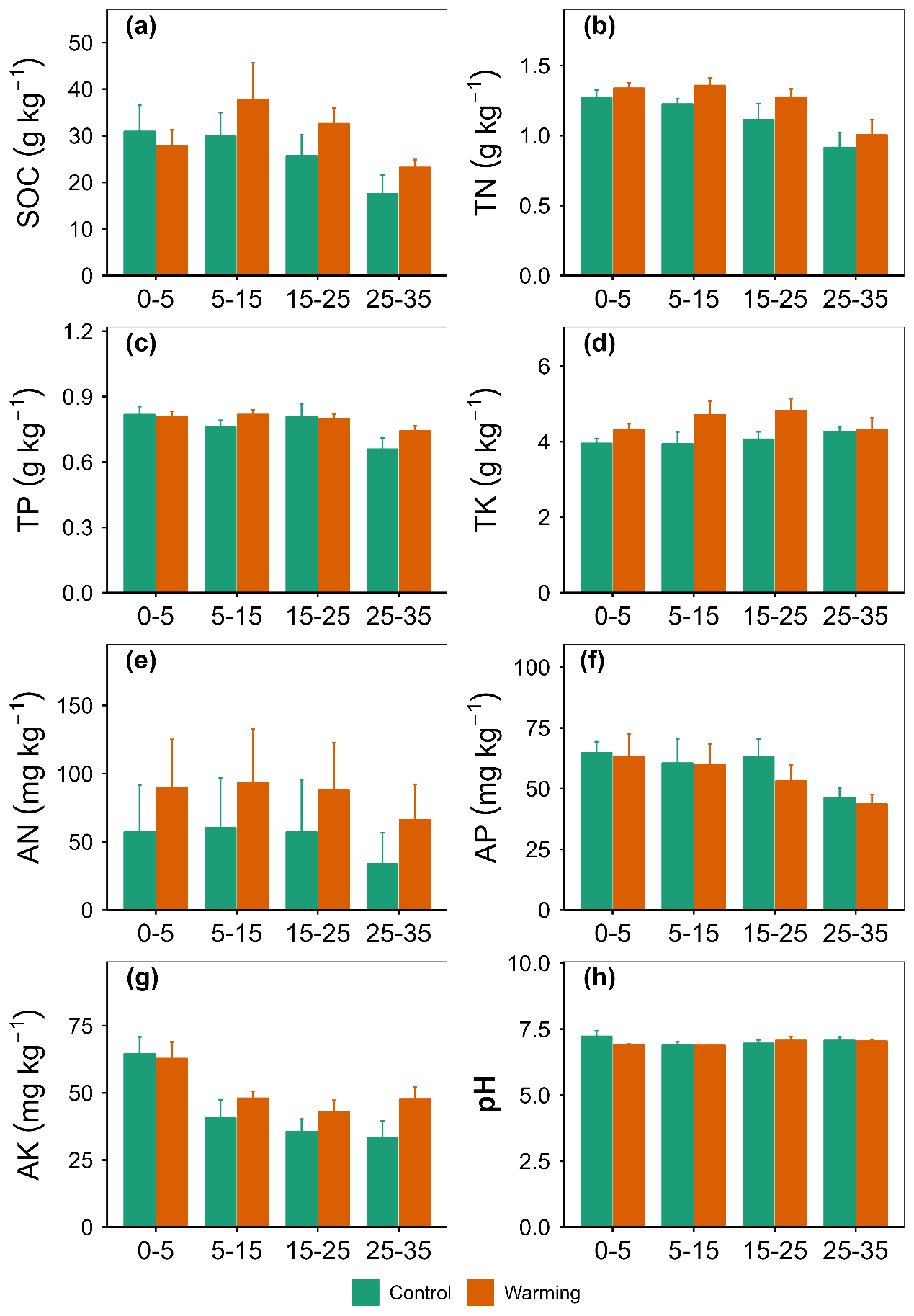


**Fig.S2**. Comparison of soil organic carbon (SOC) (a), total nitrogen (TN) (b), total phosphorus (TP) (c), total potassium (TK) (d), available nitrogen (AN) (e), available phosphorus (AP) (f), available potassium (AK) (g) and pH (h) between the control and warming treatments.
